# The role of hepatitis B virus genome variations in HBV-related HCC: effects on host signaling pathways

**DOI:** 10.3389/fmicb.2023.1213145

**Published:** 2023-07-31

**Authors:** Shahrzad Shoraka, Seyed Mahdi Hosseinian, Ayda Hasibi, Amir Ghaemi, Seyed Reza Mohebbi

**Affiliations:** ^1^Basic and Molecular Epidemiology of Gastrointestinal Disorders Research Center, Research Institute for Gastroenterology and Liver Diseases, Shahid Beheshti University of Medical Sciences, Tehran, Iran; ^2^Department of Microbiology and Microbial Biotechnology, Faculty of Life Sciences and Biotechnology, Shahid Beheshti University, Tehran, Iran; ^3^Gastroenterology and Liver Diseases Research Center, Research Institute for Gastroenterology and Liver Diseases, Shahid Beheshti University of Medical Sciences, Tehran, Iran; ^4^Foodborne and Waterborne Diseases Research Center, Research Institute for Gastroenterology and Liver Diseases, Shahid Beheshti University of Medical Sciences, Tehran, Iran; ^5^Department of Virology, Pasteur Institute of Iran, Tehran, Iran

**Keywords:** hepatitis B virus, hepatitis, chronic, carcinoma, hepatocellular, signal transduction, mutation, genotype

## Abstract

Hepatocellular carcinoma (HCC) is a significant global health issue, with a high prevalence in many regions. There are variations in the etiology of HCC in different regions, but most cases are due to long-term infection with viral hepatitis. Hepatitis B virus (HBV) is responsible for more than 50% of virus-related HCC, which highlights the importance of HBV in pathogenesis of the disease. The development and progression of HBV-related HCC is a complex multistep process that can involve host, viral, and environmental factors. Several studies have suggested that some HBV genome mutations as well as HBV proteins can dysregulate cell signaling pathways involved in the development of HCC. Furthermore, it seems that the pathogenicity, progression of liver diseases, response to treatment and also viral replication are different among HBV mutants. Understanding the relationship between HBV genome variations and host signaling pathway alteration will improve our understanding of the molecular pathogenesis of HBV-related HCC. Furthermore, investigating commonly dysregulated pathways in HBV-related HCC is necessary to discover more specific therapeutic targets and develop more effective strategies for HCC treatment. The objective of this review is to address the role of HBV in the HCC progression and primarily focus on the impacts of HBV genome variations on HCC-related signaling pathways.

## 1. Introduction

Hepatocellular carcinoma (HCC) is the most common type of primary liver malignancy and the second leading cause of cancer-related death worldwide. HCC has become a global health challenge (Galle et al., 2018; Stella et al., 2022). The frequency pattern of HCC shows an obvious geographical imbalance, with the highest incidence observed in East Asia and sub-Saharan Africa, together accounting for about 85% of all cases. In contrast, Europe, with the exception of southern Europe has a lower incidence of HCC (Galle et al., 2018).

HCC has many known risk factors and co-factors that contribute to its pathogenesis. These factors can be grouped into several categories including patients factors (male gender, elderly, and ethnicity of Asian or African), comorbidities [liver cirrhosis, metabolic syndrome, diabetes, obesity, non-alcoholic fatty liver disease (NAFLD), and non-alcoholic steatohepatitis (NASH)], environmental factors (alcohol abuse, tobacco use, and exposure to aflatoxin (AFB-1), aristolochic acid, arsenic, vinyl chloride, and other chemical carcinogens), genetic background (hereditary hemochromatosis, Wilson’s disease, α1-antitrypsin deficiency and family history of HCC) and chronic infection with hepatitis B and C viruses (Yang et al., 2019; Suresh et al., 2020; Russo et al., 2022; Stella et al., 2022).

Although alcohol-and NASH-related HCC are increasing, chronic viral hepatitis is one of the strongest risk factors for HCC worldwide (Russo et al., 2022). In most cases HCC is caused by long-term chronic hepatitis and liver cirrhosis caused by HBV or hepatitis C virus (HCV) infection (Yang et al., 2019). Although the association of hepatitis D virus (HDV) with cancer is less clear, it has been suggested to increase the risk of HCC in HBV carriers (Galle et al., 2018; Jindal et al., 2019; Trad et al., 2022). Because of this strong association between viral hepatitis and HCC, the global incidence of HCC reflects the prevalence of these viral infections (Jindal et al., 2019). While HCV is the main cause of HCC in North America, Europe, Japan, parts of Central Asia including Mongolia, and North Africa and the Middle East, especially Egypt, chronic HBV infection is the main cause of HCC in East Asian countries and most African countries, except for North Africa (Yang et al., 2019).

**Figure 1:**
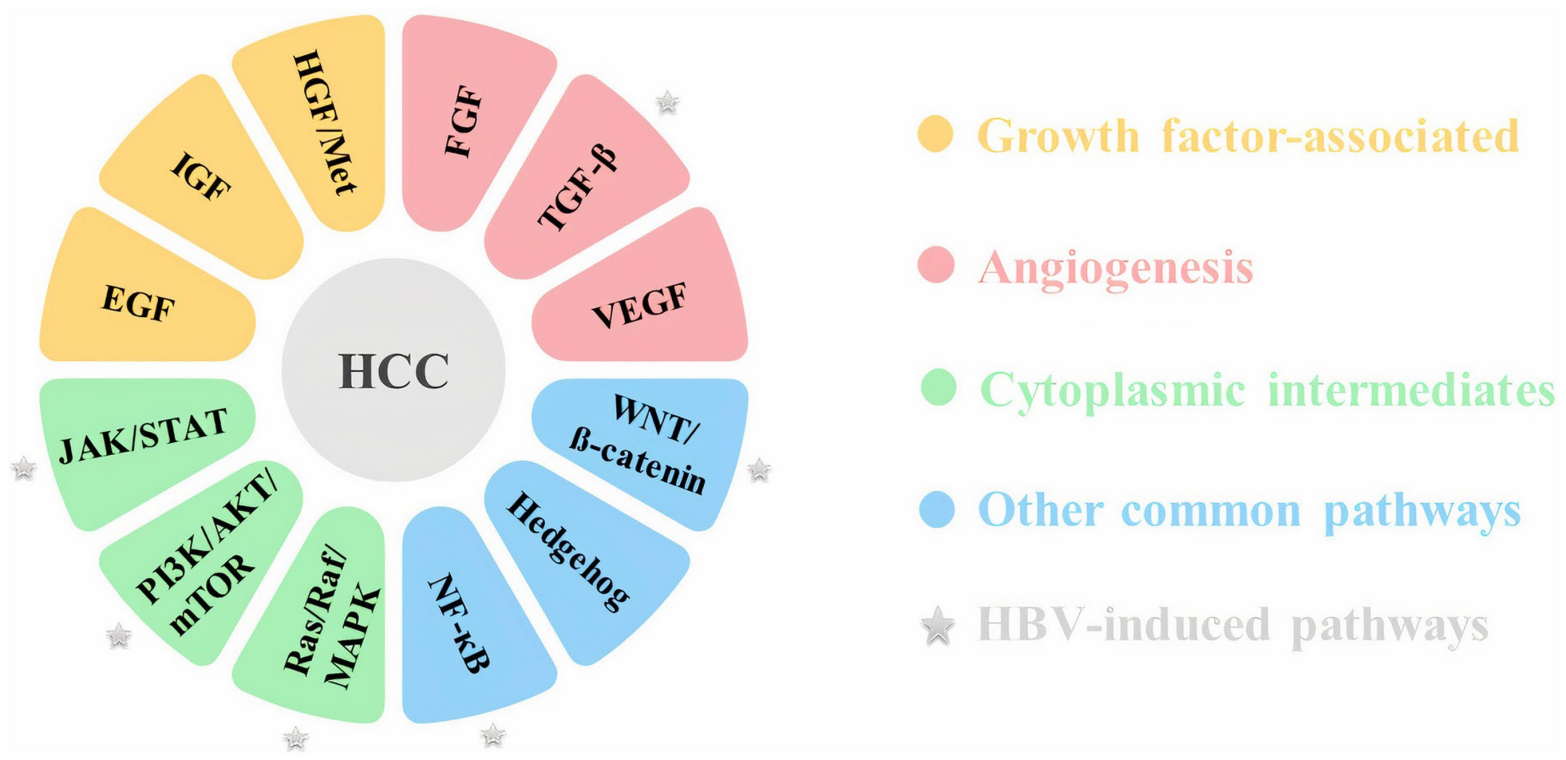
Major signaling pathways in HCC.

Despite recent advances in molecular biology techniques, the molecular pathogenesis of HCC has not yet been fully characterized (Alqahtani et al., 2019). The development and progression of HCC is a complex multistep process that may involve epigenetic alterations in addition to genetic factors (Dimri and Satyanarayana, 2020). Inactivation of tumor suppressor genes, including p53 and pRb, and activation of proto-oncogenes including Ras, c-myc and c-fos, telomere shortening, single nucleotide variants, small deletions and mutations in TERT (telomerase reverse transcriptase), APC (adenomatous polyposis coli), and CTNNB1 (catenin beta 1) genes are among the most common types of molecular disorders in HCC. Inflammation and oxidative stress, which are mainly induced by the mentioned risk factors, could lead to HCC due to induced genomic instability and activation of multiple oncogenic signaling pathways (Alqahtani et al., 2019; Suresh et al., 2020; Garrido and Djouder, 2021). Aberrant activation of some molecules involved in different signaling pathways, such as the Wnt/β catenin, PI3K/AKT, and MAPK pathways, could cause HCC development. These pathways are often related to cell cycle control, proliferation, differentiation, cell survival and apoptosis (Alqahtani et al., 2019). Major signaling pathways linked to HCC are mentioned in Figure 1.

Also, the factors related to the etiology of HCC, such as chronic viral hepatitis infections, can have a direct effect on the progression and outcome of the disease (Jindal et al., 2019). Since the molecular mechanisms underlying HCC development are complex and extensive, understanding the mechanisms involved could provide new insights into HCC management, independent of or related to the etiology of liver disease (Galle et al., 2018; Stella et al., 2022).

Considering that HBV is one of the most important causes of HCC, this study has addressed the role of HBV in HCC progression and primarily focused on the impacts of HBV genome variations on HCC-related signaling pathways.

### 1.1. Hepatitis B virus

Advanced liver fibrosis and cirrhosis is the most important risk factor (80–90% of cases) for HCC progression, and viral hepatitis is also considered an important cause of liver cirrhosis (Jindal et al., 2019). About 80% of HCCs are associated with chronic hepatitis B virus (HBV) or hepatitis C virus (HCV) infections. HBV accounts for 50–80% of virus-related HCCs, while HCV is responsible for 10–25% of reported cases (Galle et al., 2018; Jindal et al., 2019; Trad et al., 2022).

Studies have shown that the risk of HCC in HBV-infected patients is 20-fold higher compared to uninfected cases (Trad et al., 2022). HBV infection is known as the most common chronic viral infection worldwide. The World Health Organization (WHO) has estimated that approximately one-third of the world’s population has been infected with HBV at some point of time in their life. Exposure to HBV in most of these individuals has occurred in adulthood and resulted in a self-limiting infection [acute hepatitis B (AHB)] that was successfully controlled and or cleared. Less than 5% of immunocompetent adults develop persistent infection [chronic hepatitis B (CHB)], and most cases of chronic infection are acquired in infancy or early childhood. More than 250 million people in the world have CHB and around 1 million die each year from complications of chronic infection, liver cirrhosis, and hepatocellular carcinoma. CHB has high prevalence rates in Africa and Asia, as well as parts of Central and Eastern Europe (Iannacone and Guidotti, 2022). In contrast, the prevalence in most European countries is low and estimated at 0.5–0.7% (Stella et al., 2022).

Hepatitis B virus (HBV) is an enveloped DNA virus that is a member of the Hepadnaviridae family. The HBV genome is approximately 3.2 kb that forms a relaxed circular DNA (rcDNA) with a complete (−) strand and an incomplete (+) strand (Tsukuda and Watashi, 2020). The complete negative strand (L or long strand) is complementary to all HBV mRNAs, and the positive strand (S or short strand) is variable and contains 50–75% of the length of the long strand (Shi and Li, 2023).

**Figure 2:**
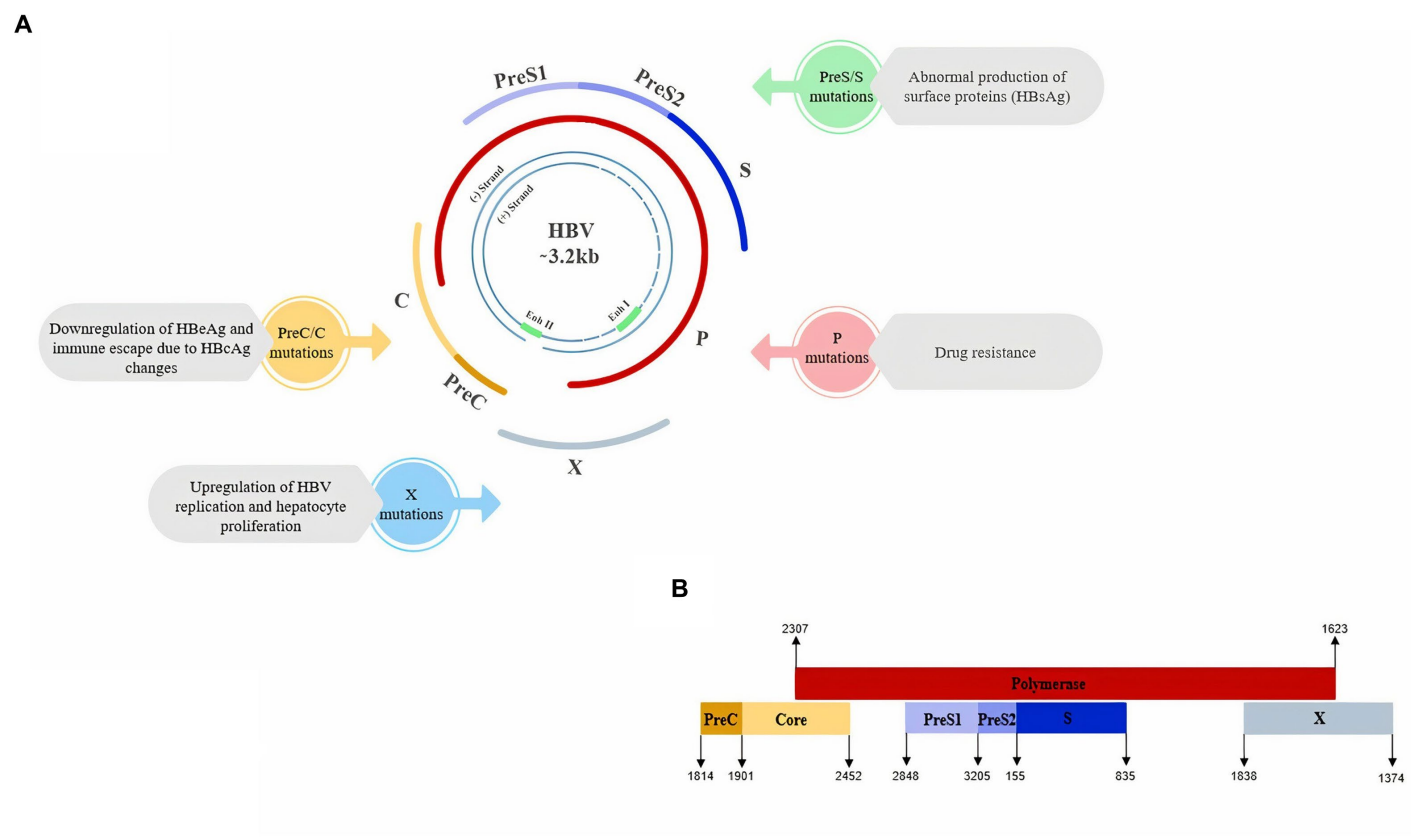
**(A)** Schematic view of Hepatitis B Virus genome and the main impacts of ORFs mutations on HBV which could lead to higher risk of HCC. **(B)** Schematic view of the overlapping pattern of HBV ORFs (Genebank MT591274.1).

The genome of HBV consists of four overlapping open reading frames (ORFs including P, preS1/S2/S, preC/C, and X) that encode major products. A polymerase protein (P) contains the domains of DNA polymerase, reverse transcriptase and RNase H. The preS/S region encodes the three forms of surface proteins [L/preS1, M/preS2, and S (HBsAg)]. The preC/C region is responsible for encoding HBcAg and HBeAg protein and including preC and C genes. X ORF encodes HBx, which has been shown to have pleiotropic functions, such as regulation of viral transcription and oncogenic activity (An et al., 2018; D'souza et al., 2020; Jiang et al., 2021; Wei and Ploss, 2021). HBV ORFs are under the transcriptional control of four promoters and two enhancers including preS1 promoter, preS2/S promoter, enhancer I/X promoter and enhancer II/basal core promoter (BCP; D'souza et al., 2020; see Figure 2).

Upon hepatocyte infection, rcDNA is released and then covalently closed circular DNA (cccDNA) is formed. cccDNA is the transcription template for HBV mRNAs. In fact, cccDNA is a stable mini-chromosome that is like an intracellular seed for initiation of the viral life cycle (Tong and Revill, 2016). HBV specifically infects hepatocytes, and since it is non-cytopathic, it leads to liver disorders through immune-mediated mechanisms (Iannacone and Guidotti, 2022). As mentioned, liver cirrhosis and HCC are not caused by acute HBV infection. But frequent cycles of hepatocyte destruction-regeneration during the immune clearance of CHB could lead to these consequences (Tong and Revill, 2016). Besides these indirect mechanisms, the hepatitis B virus could lead to malignancy through direct mechanisms (Wang et al., 2021).

## 2. Molecular mechanisms of HBV-related HCC progression

The tumorigenic activity of HBV proteins such as HBx in mouse models has characterized HBV as an oncogenic virus. However, oncoviruses provide only a part of oncogenic alterations, and in order to cause cancer, an interplay between viral and other factors (such as environment, host, and time) is needed (D'souza et al., 2020; Jiang et al., 2021; Russo et al., 2022). Therefore, in addition to viral proteins that could cause oxidative stress damage or dysregulation of cellular signaling pathways, damage caused by chronic inflammation due to HBV infection and the virus-immune interactions play a role in HCC development (D'souza et al., 2020).

Clinical studies have also suggested that high HBV DNA load, positive HBeAg, longer infection duration, co-infection with HDV, HCV, or HIV and specific HBV genotypes and genome mutations can also affect the HCC risk and progression (Levrero and Zucman-Rossi, 2016; Stella et al., 2022). For example, HBV/HDV co-infection increases the risk of HCC 2–6 fold in CHB patients and also accelerates the HCC progression (Sagnelli et al., 2020). Because HDV is a defective virus and must depend on HBV to replicate, it is unlikely that HDV directly causes HCC. It suggested that the interaction between HDV and HBV increases the HCC risk by aggravating liver fibrosis and cirrhosis. However, recent studies have shown that L-HDAg may lead to inflammation, the effects of which include endoplasmic reticulum (ER) stress and necrotizing inflammation, as well as a possible increase in the production of reactive oxygen species (ROS), which may ultimately contribute to the development of HCC (Koh et al., 2019; Puigvehí et al., 2019; Alfaiate et al., 2020). This synergistic molecular mechanism could explain why HBV/HDV co-infection is associated with a higher incidence rate of HCC compared with HBV mono-infection (Koh et al., 2019).

In the following, we briefly discussed the role of each viral factor in the progression of HBV-related HCC and also mentioned the molecular mechanisms used by HBV to promote HCC.

### 2.1. HBV proteins

#### 2.1.1. HBx

HBx is considered a key viral protein in HBV-induced carcinogenesis through versatile mechanisms. HBx contributes to HCC progression through various mechanisms, including integration of the HBx gene into the genome of hepatocytes and genomic instability, interaction with mitochondria and other cellular proteins to promote oxidative stress, cell survival signaling, tumor suppressors inactivation and epigenetic changes such as DNA and histone modifications and microRNAs expression (Shlomai et al., 2014). In addition, studies have shown that HBx can lead to increased hepatocyte transformation through interaction with cellular proteins that play a role in some tumor-related activities, DNA repair, autophagy, and cell reproduction (Geng et al., 2015). HBx could also alter the expression pattern of many host genes and trigger many signaling pathways in hepatocytes, which are involved in the control of transcription, cell cycle, proliferation and apoptosis (Levrero and Zucman-Rossi, 2016). HBx is also a multifunctional regulator to modulate various cancer-related cellular pathways. HBx could mediate protooncogenic signaling pathways, including NF-κB, JAK–STAT, MAPK/Ras/Raf/c-Jun, Src, protein kinase C, and PI3K (D'souza et al., 2020). In primary chronic hepatitis B, HBx also switches TGF-β signaling from tumor suppressor to oncogenic (Murata et al., 2009). HBx expression has been reported to be associated with β-catenin activation in 80% of HBV-related HCCs (Ding et al., 2005). Furthermore, HBx stimulates viral gene expression and replication through Smc5/6 decay and therefore, could lead to increased HBV viral load and exacerbation of CHB-induced inflammation and carcinogenesis (Decorsière et al., 2016).

#### 2.1.2. HBsAg

Oxidative stress in the endoplasmic reticulum (ER) and mitochondria can be mediated by HBsAg (Ivanov et al., 2017). During the HBV replication, the accumulation of surface proteins in the ER causes stress and thus contributes to the progression of HCC (Chaturvedi et al., 2019). HBsAg has effects on mitochondrial function and can reduce the activity of enoyl coenzyme A hydratase, short chain, 1 (ECHS1). On the one hand, this can lead to cell apoptosis through the reduction of the mitochondrial membrane potential, as well as the reduction of serine/threonine protein kinase (Akt) phosphorylation in the cell. On the other hand, it increases reactive oxygen species (ROS) formation (Stella et al., 2022). HBsAg also could cause immune dysregulation, positive regulation of survival signaling pathways, modification of transcription factors (NF-κB, STAT3), increased mutation via production of free radicals, dysregulation of the cell cycle, pro-inflammatory cytokines production and stellate cells activation which promote cell transformation (D'souza et al., 2020).

#### 2.1.3. HBeAg

Epidemiological studies confirmed the relationship between HBeAg and the higher risk of HCC. Although the pathophysiological role of HBeAg in oncogenesis remains unclear. HBeAg appears to induce cell proliferation via a G1/S transition. Studies suggested that HBeAg-mediated upregulation of miR-106b has a role in the HBV-related HCC pathogenesis by targeting the RB gene (Yang et al., 2002; Samal et al., 2017; Stella et al., 2022). Previous studies have shown that cytosolic HBeAg can inhibit IL-1β-mediated NF-κB activation and disrupt JAK–STAT signaling leading to suppression of interferon action, which ultimately increases the persistence of infection (Elizalde et al., 2021).

#### 2.1.4. HBcAg

The role of HBcAg in HCC is not very clear. Activation of NLRP3 inflammasome, release of IL-1β and activation of caspase-1 and NF-kB by HBcAg have been observed in HepG2 cells (Ding et al., 2019). It has been suggested that HBcAg may facilitate HBV proliferation by reducing IFN and MxA protein (Yang G. et al., 2022). In addition, HBcAg has shown a suppressive effect on the expression of interferon-induced membrane protein 1 (IFITM1) in HepG2 cells, which promotes HBV replication (Li et al., 2019).

### 2.2. HBV DNA integration

One of the important molecular mechanisms in the HCC is the viral DNA integration into the host genome. The only hepatotropic DNA virus that utilizes DNA integration to promote genome instability is HBV (D'souza et al., 2020). Although HBV DNA integration is not essential for viral replication, it has been reported in around 85 to 90% of tumor cells obtained from HCC cases. HBV integration appears to cause HCC by several mechanisms, including genome instability, mutations in proto-oncogenes and tumor suppressors, and expression of mutant viral proteins including altered or truncated HBsAg, HBcAg and HBx (Tu et al., 2017). HBV DNA integration at specific genomic loci could lead to the production of more mutations and cause malignant transformation in integrated cells. HBV DNA integration into host cells may be responsible for the high copy number variations (CNV) near the integration points, leading to instability of the genome, mutation induction, chromosomal deletion, and gene rearrangement of hepatocytes. Analyses have shown that HBV DNA fragments readily integrate into so-called fragile genomic regions, such as intergenic regions, repetitive regions, CpG islands, and telomeres, as well as functional regions such as genes related to cell metabolism, survival and regulation of cell cycle. Although HBV DNA sequences that integrate into the host genome include X, C, enhancer and S, in HCC patients, X and C genes are the most commonly reported integrated genes. It demonstrated that the target genes for HBV integration are significantly involved in cancer-related pathways. Also, a correlation between HBV DNA integration with alterations in tumor suppressor genes, mutation in p53 and genomic instability has been observed in HCC cases with occult HBV infection (Mak et al., 2020; Jiang et al., 2021; Pollicino and Caminiti, 2021).

### 2.3. HBV-miRs

In addition to host miRNAs, recently, it has been found that the HBV encodes miRNAs such as HBV-miR-3 that have roles in disease progression. HBV-miR-3 is encoded from nucleotides 373–393 in the HBV genome and is generated from three HBV mRNAs, PreC, PreS1, and PreS2 (Jasim, 2021). HBV-miR-3 leads to the HCC progression by controlling viral replication and reducing cell apoptosis, therefore, plays a role as an oncogene in HBV-related HCC (Tang et al., 2020; Jiang et al., 2021; Morishita et al., 2021). HBV-miR-3 by binding to the PTEN mRNA, leads to a decrease in PTEN protein level and subsequently reduces apoptosis and promotes invasion and proliferation (Tang et al., 2020). On the other hand, HBV-miR-3 can activate the JAK/STAT signaling pathway in hepatocytes (Zhao et al., 2020).

### 2.4. Epigenetic remodeling

One of the pathogenesis mechanisms of HBV-related HCC is the host epigenetic changes. HBV could lead to epigenetic remodeling through altering host DNA methylation, histone modifications and non-coding RNA expression. Abnormal epigenetic remodeling, activation of oncogenes and inactivation of tumor suppressor genes can occur by HBV structural proteins (mainly HBx; Jiang et al., 2021; Zeisel et al., 2021; Yang L. et al., 2022).

### 2.5. Metabolic reprogramming

Metabolic reprogramming is known as one of the important mechanisms in the progression of malignant tumors. Although there are limited studies in this regard, it seems that dysregulation of specific liver metabolic pathways in HBV-related HCC could be attributed to viral infection. Metabolic reprogramming induced by HBV not only by affecting tumor cells but also by affecting the extracellular microenvironment may lead to the promotion of tumorigenesis (Jiang et al., 2021).

### 2.6. Dysregulation of tumor-promoting signaling pathways

**Figure 3:**
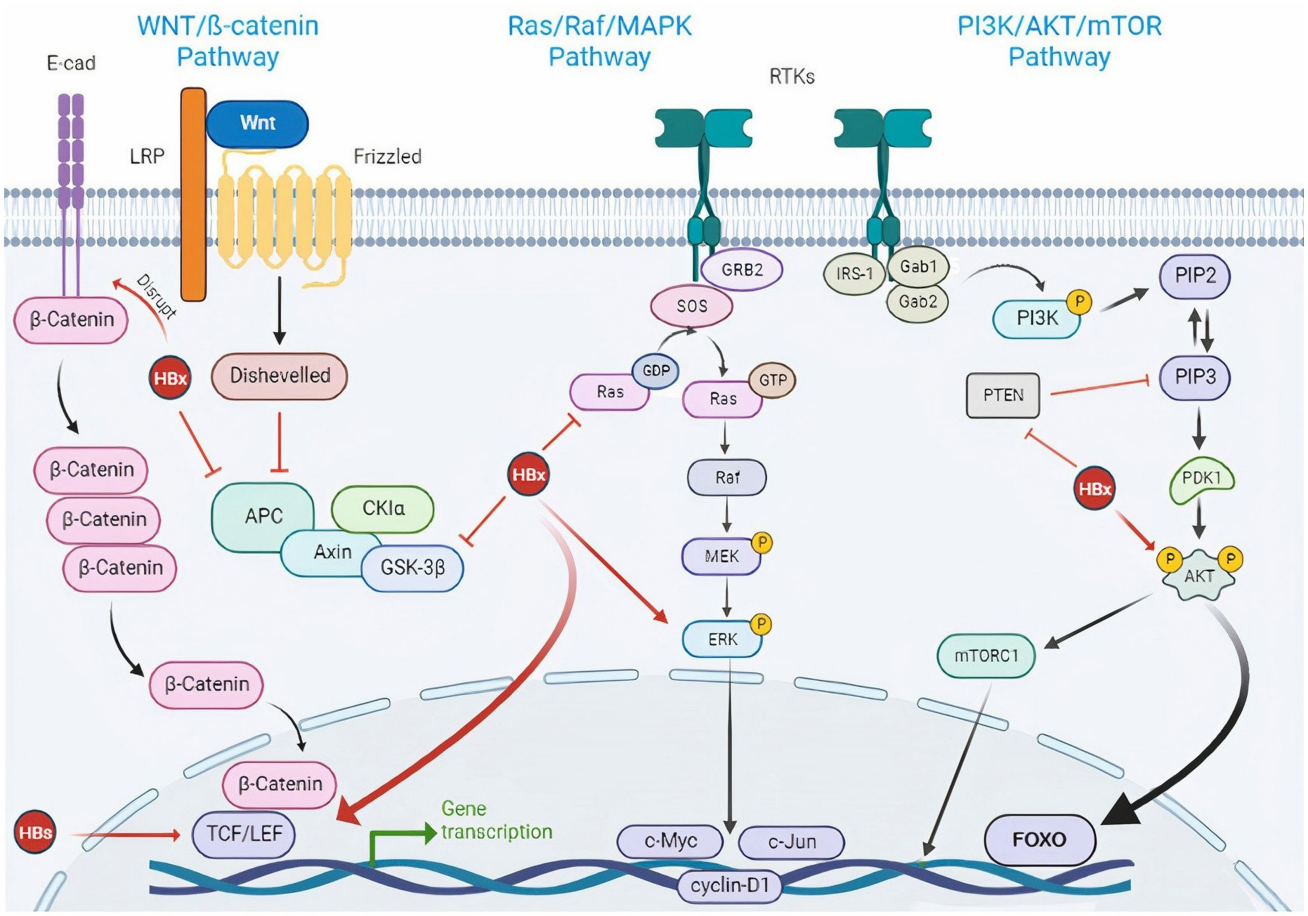
HBV-induced signaling pathways in HCC.HBV is engaged in cancer-associated signaling pathway activation. *Wnt/β-catenin signaling pathway:* Wnt is a glycoprotein ligand that activates a receptor-mediated signaling cascade by acting as a ligand for the Frizzled cell surface receptor family. In most cells, β-catenin binds to E-cadherin when Wnt ligands are not present. Casein kinase 1a (CKIa) and glycogen synthase kinase 3β (GSK3β) are activated and continuously phosphorylate and degrade cytosolic β-catenin through complex formation between cytosolic β-catenin and adenomatous polyposis coli (APC) and AXIN. When the Wnt ligand is present, the extracellular Wnt ligand interacts with the FZD/LRP5/6 receptor complex on the cell surface. This causes GSK3β to be phosphorylated and inhibited, which results in an accumulation of cytosolic β-catenin. The transcription of target genes such as cyclin D1, c-Myc, and c-MET is then activated as a result of β-catenin’s translocation to the nucleus and interactions with TCF and LEF transcription factors which can be activated by HBx and HBs. Again, the HBx protein can inhibit E-cadherin to activate the Wnt pathway. On the other hand, because of its capacity to bind with APC, HBx can prevent the degradation of β-Catenin. Besides, the Wnt signaling pathway becomes unusually activated when the GSK3β degradation complex is blocked by HBx. *PI3K/AKT/mTOR signaling pathway:* IRS-1 and adaptor proteins (Gab1/2) binding activate PI3K in the PI3K/AKT/mTOR signaling cascade. PIP3 (phosphoinositol triphosphate), a second lipid messenger produced by PI3K, then activates the serine/threonine kinase AKT. mTOR is one of the cytoplasmic proteins that is phosphorylated by activated AKT, which also controls a number of transcription factors. HBx can inhibit PTEN, a suppressor of PIP3 and also activate Akt directly. *Ras/Raf/MAPK signaling pathway*: The Ras/Raf/MAPK signaling pathway is triggered by ligand binding and phosphorylation of several growth factor tyrosine kinase receptors, including activation of the Grb2/SOS adaptor complex and downstream activation of mitogen/extracellular protein kinase MEK. ERK, the downstream signaling molecule, is then phosphorylated by MEK. Numerous substrates that affect the cytoplasm and nucleus are used by ERK1/2 to control cellular activity. HBx can activate Ras and ERK directly. E-cad, E-cadherin; LRP, low-density lipoprotein receptor-related protein; LEF, lymphoid enhancer-binding factor; TCF, T cell factor; FZD, frizzled; RTK, receptor tyrosine kinase; HBx, hepatitis B X protein; HBs, hepatitis B surface antigen; HBV, hepatitis B virus; Glycogen Synthase Kinase 3 (GSK-3β); Adenomatous Polyposis Coli (APC), Casein Kinase 1a (CKIa); PIP3, phosphoinositol triphosphate; PIP2, phosphoinositol diphosphate; IRS-1, Insulin receptor substrate 1; Gab, GRB2-associated-binding protein; Grb2, growth factor receptor-bound protein 2; SOS, Son of Sevenless.

It has been also suggested that several signaling pathways are dysregulated in HCC, which can be in response to viral infection (Dimri and Satyanarayana, 2020). HBV can affect the key factors of oncogenic signaling pathways. The HBV structural proteins lead to the regulation of many signaling pathways and have an important role in the disease progression. Integrations of HBV DNA in the host genome, as well as HBV mutations, play an important role in the dysregulation of tumor-promoting signaling pathways (Jiang et al., 2021). It has also been shown that most of the target genes for HBV integration are enriched in cancer-related pathways, such as MAPK and Hedgehog signaling, and the integration frequency of HBV is expected to increase along with the progression from HBV infection to HCC (Yang et al., 2018; Jiang et al., 2021). HBV can lead to the abnormal activation of the Wnt/β-catenin signaling pathway. In addition, activated β-catenin could interact with other transcription factors, such as TGF and HIF-1α, leading to the regulation of target gene expression and promoting disease progression (D'souza et al., 2020). Also, HBx exacerbates HBV-associated HCC by inhibiting PTEN (as a tumor suppressor) and activating PI3K/Akt signaling pathway (Kim et al., 2021). HBV can activate the MAPK/ERK pathway. MAPK signaling has a significant role in immune evasion, promoting the survival of tumor cells and anti-cancer drug resistance. In the presence of HBV, the p38 MAPK pathway plays an important role in the secretion of HBsAg/HBeAg, viral replication, and HBV cccDNA formation (Moon and Ro, 2021). Some HBV structural proteins, such as HBx could also induce ROS production and oxidative stress. ROS has been shown to have a direct promoting role in viral replication, disease severity, and malignant transformation (Ivanov et al., 2017; see Figure 3).

### 2.7. HBV genome variations

Considering that HBV variability plays a significant role in the progression of liver disease and the effectiveness of treatment, various methods have been developed to identify viral genome mutations. Techniques based on restriction fragment length polymorphism or selective hybridization of amplified fragments have been used. Nucleotide sequencing is the gold standard method for mutation detection. In comparison to the Sanger sequencing, second-generation sequencing has improved sensitivity for detection and quantification of mutations, mixed genotypes, and viral recombination while third-generation sequencing enables the analysis of the whole virus genome (Lee et al., 2018; Rajput, 2020; Garcia-Garcia et al., 2021).

The genetic diversity of the HBV genome could be classified into two levels. The first is the infection of individuals in different geographical areas with different genotypes of HBV (A-J). The second level is viral evolution in a patient during the chronic course of HBV infection. The different types of mutations are caused in response to the host immunity or antivirals (Tong, 2005; MOHEBI et al., 2009; Tong and Revill, 2016; Al-Qahtani et al., 2020).

In addition to viral proteins such as HBx, some mutations in the HBV genome could dysregulate normal signaling pathways and contribute to HBV-related HCC progression (Wang et al., 2021). In addition to the host genetic background and somatic mutations, the mutation in HBV can also lead to an increase in the malignant transformation of hepatocytes. Mutations in the HBV gene could be caused by a variety of reasons such as long-time infection and inadequate use of NAs (nucleoside/nucleotide analogs) as well as the immune pressure of the host (Wei et al., 2017; Jiang et al., 2021). Since antiviral treatment is often prescribed for a longer duration in CHB patients, under drug selection pressure, viruses with resistance mutations will arise and propagate. Furthermore, the second-line treatment of HBV including Lamivudine, Adefovir, and Entecavir has a low genetic barrier to the development of resistance-associated mutations (Zhang et al., 2019; Nguyen and Van Le, 2023). Therefore, in order to reduce the development and spread of drug resistance, avoiding unnecessary treatment, careful selection of therapy including Tenofovir and Tenofovir alafenamide and continuous monitoring can be helpful (Shaw et al., 2006).

HBV mutants might change vital epitopes for recognition by host immunity, increase HBV replication leading to enhanced virulence, or display resistance to antivirals as well as improving cell attachment or penetration. These viral mutants have been associated with a high risk of liver diseases (Chen, 2018). Also, vaccine escape mutations in HBV may reduce the success of existing treatment and prevention strategies (Mokaya et al., 2018). Since HBV vaccination results in the production of neutralizing antibodies that mainly target the second hydrophilic loop of the “a” determinant of HBsAg, residue changes in this region of the S antigen could lead to resistance to the HBV vaccine induced immunity. Also, other HBsAg mutations (alone or in combination) that may lead to HBV vaccine escape, have been identified worldwide (Coppola et al., 2015; Colagrossi et al., 2018; Inoue and Tanaka, 2020; Mokaya et al., 2021).

## 3. HBV genotypes and impacts on HCC

The 10 known HBV genotypes (A-J) have diverged more than 8% in the whole genome sequence. The distribution of HBV genotypes in geographical locations is different. In Africa, Europe and the Americas, genotypes A and D, in Asia genotypes B and C, and in sub-Saharan Africa genotype E are common. Genotypes F and H are most commonly reported in South and Central America, genotype G in France, Germany, and the United States, genotype I in Vietnam and Laos, and genotype J in the Ryukyu Islands of Japan. Although all genotypes can lead to progressive liver disease, the clinical outcome of each genotype are different. Pathogenicity, progression of liver diseases, response to antivirals, as well as HBV replication have been reported to be different between HBV genotypes. But so far, the molecular basis or biological properties of the virus have not been attributed to these differences (Mohebbi et al., 2008; Pourkarim et al., 2010, 2014; Chen, 2018; Romani et al., 2018; Elizalde et al., 2021).

It seems that there is an association between different genotypes of HBV with distinct biology in infected populations. For example, many studies have shown that in East Asia, HBV genotype C infection is correlated with a more severe outcome of liver diseases such as cirrhosis and HCC, while in other epidemic regions, HBV genotype C, D, and F have been reported to have a greater risk of HCC (McMahon, 2009; Lin and Kao, 2015; Raffetti et al., 2016). Studies have also shown that genotypes B, C, and subgenotype A1, are associated with HCC progression (Tong and Revill, 2016). A study on a cohort of Alaska Natives followed for more than 30 years showed that cases with genotypes A, C, and F were at greater HCC risk compared to genotypes B or D (Ching et al., 2016). Another retrospective cohort study demonstrated that HBV subgenotype F1 is associated with a higher HCC risk in Alaska Native children and young adults (Gounder et al., 2016). Therefore generally the HBV genotypes associated with the highest risk of HCC are C, B, F, D, and A genotypes, respectively (D'souza et al., 2020).

Different viral replication capacities among genotypes may explain the clinical differences observed among HBV genotypes. Low replication activity of the virus may contribute to evasion of the immune response and thus persistence of infection. On the other hand, the rapid viral replication and the intracellular accumulation of replicating intermediates may cause a more intense but short-term immune clearance phase that could lead to acute liver failure (Lee et al., 2016). The results of *in vitro* studies in this regard are challenging. Some studies have reported HBV replication capacity is higher in subgenotypes B2, F1b and D1, respectively, while subgenotypes A2 and C1 have the lowest replication capacity while others have reported a higher replication rate for subgenotype A2, C2 and D3 than genotype B2 or higher capacities for genotype C in comparison to B2 and A2 subgenotype (Sugiyama et al., 2006; Qin et al., 2011; Sozzi et al., 2016; Elizalde et al., 2021). These controversial results may be due to the different experimental systems used in the studies. Although a high HBV replication rate in the hepatocyte has been reported to be associated with an increased risk of HCC progression (Shi and Shi, 2009), more studies are needed. Also, direct extrapolation of results obtained from *in vitro* models to the clinic should be done with caution.

The serum HBV viral load in cases with HBV genotype C is significantly higher than in those infected with genotype B. Long-term exposure to high levels of HBV in these patients may explain the greater risk of HCC in genotype C (An et al., 2018). Also, the HCC risk is strongly related to HBV viremia. Increased HCC-related mortality has been reported in cases with baseline HBV DNA above 10^6^ copies/mL compared to those with baseline HBV DNA below 300 copies/mL (Stella et al., 2022).

Significant differences have also been reported in the expression and secretion levels of HBeAg between different HBV genotypes. An *in vitro* study showed the highest extracellular HBeAg level for the C1 subgenotype and the lowest for D1 and F1b subgenotypes (Elizalde et al., 2021). Another study conducted on serum samples of HBV patients showed significantly higher levels of HBeAg in genotype C cases than those with genotypes B, A1 or D (Cooper et al., 2021). As previously mentioned, HBeAg has a role in the HBV-related HCC pathogenesis by targeting the RB gene, which can explain the higher HCC risk of HBV genotype C.

A study was conducted in 2019 using next-generation sequencing to compare gene expression in hepatocytes collected from two groups of human hepatocyte chimeric mice, including mice infected with HBV genotype A and mice infected with HBV genotype C. The results showed that although similar pathways including inflammation caused by chemokine and cytokine signaling pathways, the p53 and integrin signaling pathways were affected by HBV infection, the expression levels of up-regulated genes by HBV genotype A or C infection were completely different. Genes related to the p53 pathway and inflammation caused by chemokine and cytokine signaling pathways were more expressed in hepatocytes infected with HBV genotype A, while genes related to cholecystokinin receptors (CCKR) signaling map, oxidative stress response and Wnt signaling pathways were more expressed in cells infected with HBV genotype C. On the other hand, the levels of inflammatory cytokines and chemokines, including CCL2, CXCL8, CXCL9, and CXCL10, were relatively higher in HBV C genotype infection than in HBV genotype A infection. In addition, the inflammation-related pathways were strongly induced by HBV genotype C infection compared to HBV genotype A infection. They suggested that the observed differences may be related to genotype differences in clinical outcome or response to treatment (Tsushima et al., 2019). Given that the role of persistent oxidative stress and activation of the Wnt signaling pathway is well established in carcinogenesis, the results of this study provide insight into the potential mechanisms underlying genotype-specific differences in the clinical features of CHB infection.

Data-independent acquisition (DIA)-based liquid chromatography-mass spectrometry clearly showed that HBV genotypes B and C infections are associated with dissimilar protein profiles and different signaling pathways. KEGG pathway enrichment analysis showed that the proteins expressed in the serum of patients infected with HBV-B are more involved in the regulation of complement and coagulation cascades and *Staphylococcus aureus* infection, while in the serum of patients with HBV-C infection, they are mainly involved in the signaling pathways including NF-κB, PI3K-Akt, focal adhesion, ECM receptor interaction, cell adhesion, and complement and coagulation cascades (Chen et al., 2021). Pathways activated in infection with HBV genotype C are mainly associated with proliferation, inflammation and tumor cell survival, so the protein pattern observed in this study confirms the higher HCC risk of HBV genotype C.

HBV genotype G is often characterized by a lack of secretion of HBeAg and very low secretion of HBsAg. *In vitro* study showed that in cells expressing HBV genotype G compared to cells expressing HBV-A, there is an intracellular accumulation of HBsAg and impaired activity of Nrf2 (detoxification-related gene), which leads to an increase in the level of ROS. Further activation of JNK leads to IRS-1 ser-phosphorylation, which is known to impair insulin signaling, a key factor of liver regeneration. It seems that the PreS1PreS2 region of HBV-G, which is responsible for the formation of aggregates, inhibits the secretion of HBsAg in the ER, thereby reducing the transcriptional activator function of LHBs (Peiffer et al., 2015). These observations partially justify the HBV G genotype-specific pathogenesis and clinical implications.

On the other hand, many studies have investigated the relationship between HBV genotype-specific mutations with increased risk of HCC, and the role of these mutations in higher risk of HCC has been confirmed (Liu W. et al., 2022).

## 4. HBV mutations and impacts on HCC

The mutation rate for HBV has been reported in the range of 1.4–3.2 × 10^−5^ base substitutions/site/year. Since HBV genome replication requires reverse transcription, and due to the lack of proof-reading, this act may cause hyper mutation phenomena. On the other hand, the mispairing between template and replicated DNA is probably due to the action of topoisomerase or as a result of splicing, can cause mutations in the form of long deletions. Recombinant virus formation could also be due to the co-infection and circulation of different HBV genotypes (Tong and Revill, 2016; Rajput, 2020). As expected, mutations that occur naturally or during antiviral treatments have a critical role in viral latency, immune evasion, the pathogenesis of liver disorder, and resistance to antivirals (Rajput, 2020). Also, the association between core promoter mutations, preS region deletions, C-terminal truncation of envelope proteins, and spliced pre-genomic RNA has been reported with the progressive liver disease and development of HCC (Lin and Kao, 2015; Tong and Revill, 2016). In addition to core promoter mutations, mutations that lead to decreased expression of HBeAg are also involved in acute liver failure (Tong and Revill, 2016). Common HBV mutations are mentioned in Tables 1, 2.

**Table 1:** Common mutations in HBV preS/S and preC/C.

Mutation	Outcome	References
*PreS/S mutants*		
sW172stop (aa)	Generates a stop codon in the HBsAg Impairs secretion of HBsAg Reduces viral fitness Increases the risk of HCC	Sheldon and Soriano (2008), Yeh et al. (2011), Lai and Yeh (2008)
C3116T (nt) /T31C (aa) combo mutation	The frequency of the single mutation is very low compared to the combo mutation Promoting hepatocarcinogenesis by inducing ER stress, and STAT3 pathways Increases the risk of HCC in patients without antiviral therapy	Liu W. et al. (2022)
A2962G (nt) C2964A (nt)	Associated with an increased risk of HCC Higher tumor cccDNA level Also reported as A2962G/C2964A combo mutation	Wang et al. (2015)
T53C (aa)	Associated with an increased risk of HCC	Yin et al. (2010), Jiang et al. (2009), Sung et al. (2008)
C7A (aa)	Increases risk of HCC	Qu et al. (2014), Thi Cam Huong et al. (2022), Rivière et al. (2019)
C105T (aa)	Higher tumor cccDNA levels	Wang et al. (2015)
W4P/R (aa)	This male-specific mutation is significantly related to HCC	Thi Cam Huong et al. (2022), Caligiuri et al. (2016)
*PreC/C mutants*		
G1896A (nt)	*known as the most important mutation in HBV-related HCC Prevents expression of HBeAg protein Increases HCC tumor growth *in vivo* via enhancing HBV replication and activating the ERK/MAPK pathways Strongly associated with HCC	Wei et al. (2017), Zhao et al. (2023)
C1766T (nt) T1768A (nt)	Increases viral replication and viral infectivity Associated with acute liver failure and CHB progression C1766T/T1768A combo mutants were associated with higher HBV-DNA levels and increased liver disease	Liu Y. et al. (2022), Kitab et al. (2012), Dong et al. (2008)
A1762T (nt) or K130M (aa) G1764A (nt) or V131I (aa)	Contributes to an inefficient immune response that leads to hepatocarcinogenesis A1762T/G1764A mutants are associated with the increased HCC risk	Wei et al. (2017)
T1753V (nt)	Increases risk of HCC Associated with severe clinical outcome Linked with enhanced viral replication and/or reduced HBeAg expression and related with acute liver failure in some instances	Kitab et al. (2012), Parekh et al. (2003)
C1914G (nt)	Associated with HCC	Asim et al. (2010)

(nt), for nucleotide change/name; (aa), for amino acid change/name.

**Table 2:** Common mutations in HBV P and X region.

Mutation	Outcome	Reference
*P mutants*		
rtA181T (aa)	Increases risk of HCC rtA181T/sW172stop mutants have been reported in Adefovir and Lamivudine-resistamt patients. It is also associated with HCC	Yeh et al. (2011), Lai and Yeh (2008), Liang et al. (2013)
sF220L (aa)	Reduces HBsAg secretion and promotes cell apoptosis	Choi et al. (2019)
*X mutants*		
A/G1479C/T/G (nt) or T36A/S/P (aa)	It increases the viral genome integration in the host cell, which leads to insertion mutations and a 3′-terminal truncation of HBx	Xie et al. (2014)
G1521A/C (nt) or G50R (aa)	Association with HCC	Tuteja et al. (2014), Cho et al. (2011)
C1653T (nt) or H94Y (aa)	Enhancing the transcriptional activity of HBx protein Promotes HCC malignancy by altering the ROS level and some cytokines	Zhang et al. (2022)
G1613A (nt)	May induce hrpatocarcinogenesis	Tatsukawa et al. (2011)
T1674C/G (nt)	Risk factor for HCC development	Zhou et al. (2022), Xie et al. (2013)

(nt), for nucleotide change/name; (aa), for amino acid change/name.

### 4.1. PreS/S mutations

PreS mutations, particularly preS1 deletion, preS2 deletion, and preS2 start codon mutations, may cause abnormal production of surface proteins (Levrero and Zucman-Rossi, 2016; An et al., 2018). Deleted or mutated HBsAg is associated with a greater risk of HCC. These mutations frequently appear in patients with CHB, ranging from 6% in primary infection to 60% in patients with HCC (Wang et al., 2006). Pooled relative risk (RR) of HCC for preS/S deletions and preS2 start codon mutation was reported to be 4.0 and 2.6, respectively (Yang et al., 2015). Also, the potential oncogenic properties of these mutated HBV surface proteins and the ability to induce dysplastic nodules have been demonstrated in transgenic mice (Choi et al., 2019; Wang et al., 2021).

Deletion in preS in integrated HBV DNA may impair HBsAg secretion, which also leads to increased ER and oxidative stress in hepatocytes (Liang et al., 2013). Different preS mutations lead to the activation of different mechanisms of ER stress in hepatocytes through the accumulation of LHBs proteins (Li et al., 2016). Misfolding PreS/S proteins accumulated in the endoplasmic reticulum lumen could lead to ER stress, thereby inducing oxidative damage of DNA and genomic instability (Wang et al., 2006). Alternatively, in a stress-independent way, PreS2 mutant can induce the decay of RB and p27 through interaction with c-Jun activation domain-binding protein 1 (JAB1; Wang et al., 2012; Hsieh et al., 2013) or increase the expression of cyclooxygenase-2 (COX-2) and cytoplasmic cyclin A, which contributes to the oncogenic process. The upregulation of the cyclin A pathway could increase chromosome instability through centrosome duplication and promotes hepatocyte proliferation (Wang et al., 2012, 2021). The accumulation of preS mutants in ‘ground glass’ hepatocytes has been characterized (Su et al., 2014). PreS1 mutant also leads to activation of higher levels of ER chaperones (Grp78 and 94), calcium release, and inflammatory cytokines which lead to apoptosis (Su et al., 2014). Also, the hepatocarcinogenic effects of HBV preSl mutations have been reported through TGF-α gene transactivation (Li et al., 2016). In addition, PreS2/S mutant proteins can transcriptionally activate TERT expression (Rizzo et al., 2022).

Furthermore, stop codon mutations in the S region promote tumor growth by encoding truncated proteins with transcriptional transactivation activity. In addition, premature stop codon mutations at position 172 or 182 in the S region are associated with an increased risk of liver cirrhosis and HCC (Lai et al., 2009; Pollicino et al., 2014).

The risk of HCC is significantly increased in patients without antiviral treatment with G2950A/G2951A/A2962G/C2964A and C3116T/T31C mutations, while preS2 deletion leads to an increased risk of HCC in patients receiving antiviral treatment. preS2 deletion and G2950A/G2951A/A2962G/C2964A, C3116T/T31C can promote hepatocarcinogenesis through induction of ER stress, metabolism alteration and pro-oncogenic inflammatory pathways of STAT3 (Liu W. et al., 2022).

F141L preS2 mutants have increased the risk of HCC in patients with HBV genotype C infection (Mun et al., 2011). An *in vitro* study determined that F141L-LHBs can induce cell cycle progression by downregulating p53 and p21 pathways and upregulating cyclin-dependent kinase 4 and cyclin A. It has been suggested that F141L-LHBs may play a critical role in the pathogenesis of HCC by inducing cell proliferation and transformation (Chen, 2018).

In addition, S nonsense mutations such as sC69*, sL95*, sW182*, and sL216* were detected in HCC tumors. Most of these mutants, especially sW182*, had higher cell proliferation activities and transformation abilities than wild type S (Huang et al., 2014).

The role of HBV preS1 W4P mutation in the HCC development in CHB men has been suggested in an IL-6-dependent manner. IL-6 levels in HCC patients infected with W4P mutant were significantly higher than those with wild type. This indicates the direct involvement of HBV LHBs in the gender disparity of tumorigenesis (Lee et al., 2015).

### 4.2. HBx mutations

The HBV X region consists of important *cis-*regulatory elements including enhII, CP and microRNA binding region (Zhang et al., 2022). A higher ability to promote HBV replication and increase hepatocyte proliferation of some HBx mutants was confirmed *in vitro* compared to wild-type HBx (Lin et al., 2019). Also, a greater risk of HCC has been demonstrated in HBV-related HCC clinical samples associated with several mutations in HBx (Lee et al., 2011). There is also a positive correlation between the number of HBx mutations and HCC risk (An et al., 2018). Genetic analysis has shown that there are two essential types of HBx gene variants in HBV-related liver disease: first, several point mutations in the HBx gene and second, C-terminal truncated HBx mutants (Xu et al., 2019). HBx mutations, especially C-terminal truncated mutants, are often observed in HCC tumor tissues and rarely in the surrounding non-tumor tissues of the patients (Wang et al., 2004). Also, several studies have suggested the role of C-terminal truncated HBx expression in liver carcinogenesis (Ma et al., 2008; Rivière et al., 2019). Transcriptional and anti-proliferative activity have been lost in C-terminal truncated HBx variants and they have acquired new properties such as promoting cell growth and cooperating with oncogenes in cell transformation (Rivière et al., 2019). These HBx mutants can increase the transforming abilities of Ras and Myc (Tu et al., 2001). Furthermore, the result of the study conducted in 2016 showed that the oncogenic role of C-terminal truncated HBx may be greater than wild type HBx. They attributed this to the inhibition of tumor suppressive β-catenin/ bone morphogenetic protein and activin membrane-bound inhibitor (BAMBI) signaling by C-terminal truncated HBx (Lee et al., 2016). C-terminal deletions mutants of HBx also lead to the induction of centromere protein A (CENP-A) expression through an unknown mechanism, which has been shown to be associated with HCC progression (Liu et al., 2012).

Studies have shown that the first 50 amino acids in the N-terminal sequence of HBx are necessary and sufficient for cell transformation. However, the C-terminal domain may have a role in cell proliferation and tumor development. The identification of HBx inactivating mutations in more than 70% of HBV-related HCCs can indicate the role of HBx in the progression and not in the maintenance of HCC (Amaddeo et al., 2015; Rivière et al., 2019; Wang et al., 2021).

HBx mutations including AG1762/1764TA (KV130/131MI), C1653T (H94Y), C1485T (P38S), T1753C (I127T), A1383C, and G1613A, with or without substitution of amino acids, probably alter HCC survival (Zhang et al., 2022). Some mutations, such as C1653T, T1753V, and A1762T/G1764A (TA), can contribute to HCC by altering the binding ability of several nuclear factors or amino acid sequence of HBx. A1762T/G1764A mutant also leads to an increase in the capacity of HBV replication and suppression of HBeAg serum level *in vitro* (Liu et al., 2009).

C1653T mutation showed a significant increase in the promotion of HBx-induced proliferation, invasion and migration. Also, the C1653T mutant significantly enhanced apoptosis in HCC cells. The C1653T mutation also had a promoting impact on the tumor growth effect of HBx in an HCC xenograft mouse model. It seems that C1653T mutation leads to HCC by promoting fibrosis and intracellular ROS production and changing the expression of monocyte chemotactic protein 1 (MCP-1) and interleukin 18 in the HepG2 cell line (Zhang et al., 2022). T1753V could affect apoptosis by altering the binding capacity of HBx with bcl2 (Geng et al., 2012). A study conducted on Indian CHB patients with genotype A or D showed that the T1753V mutant is often associated with advanced liver disease and leads to an increased risk of HCC (Tuteja et al., 2014).

Non-cirrhotic CHB patients with triple mutations, HBx5 + HBx130 + HBx131, have a high risk of developing HCC. Also, these triple mutations compared to HBx130 + HBx131 double mutations show a greater risk of HCC in CHB patients. HBx5 mutation in the C2 HBV genotype has been characterized as a risk factor for HCC development. Meanwhile, higher NF-κB activity has been reported in Huh7 cells with HBx5 mutation and double mutation compared to Huh7 cells with triple mutant or wild type (Lee et al., 2011).

HBx Combo mutant (T1753A/A1762T/G1764A/T1768A) upregulates s-phase kinase-related protein 2 (SKP2) via activation of the Rb/E2F1 pathway, thereby promoting the degradation of p21 and p53. Also, the Combo mutant enhances cell proliferation by increasing the fraction of cells in the S phase and the amount of DNA synthesis (Yan et al., 2015; Chen Z. et al., 2016). Furthermore, this mutant greatly abolished the activity of NF-κB and impede the HBx pro-apoptotic effects (Li et al., 2013).

### 4.3. PreC/C mutations

As mentioned, the HBeAg production and core protein are regulated by the core promoter/precore (CP/PC) region. The HBV CP/PC region is a mutation hot spot and there is a close relationship between these mutations and the clinical outcome and severity of CHB (Lin and Kao, 2017). Also, the HBV core promoter region overlaps with the C-terminal coding sequence in the HBx gene and mutation in this region may lead to increased carcinogenesis by changing the biological functions of HBx (Chen L. et al., 2016).

The G1896A stop codon mutation in the preC region results in HBeAg translation stopping prematurely (Mohebbi et al., 2012; Tsuge, 2021). A recent study showed that G1896A mutation could promote HCC tumor growth *in vivo* by enhancing HBV replication and activating of ERK/MAPK pathway (Zhao et al., 2023). Also, a natural double mutation in the basal core promoter (BCP), A1762T/G1764A (TA), has been suggested as a risk factor for HCC (Yang et al., 2016). Since the BCP region partially overlaps with the X region, the same nucleotide changes lead to K130M and V131I in HBx and A1762T/G1764A in BCP (Lee et al., 2011). The double mutation in BCP decreases the production of HBeAg and viral load before HBeAb seroconversion. It has been suggested that the oncogenic mechanism of this mutation is not directly attributable to the viral replication level (Fang et al., 2009; An et al., 2018). In addition, A1762T/G1764A (TA) double mutation along with other mutations [TA-combined mutations (TACO)] and activated AKT have been reported to be more common in poor HCC. Phosphorylated AKT (pAKT) and TACO have a critical role in HCC progression. Also, the core promoter region mutations regulate the most critical downstream effector of AKT, SKP2, thereby downregulating p21, leading to accelerated cell cycle, cell proliferation and anchorage-independent cell growth (Chen L. et al., 2016). Also, P5H/L/T mutation leads to hepatocellular carcinoma by inducing ER stress and ROS production (Kim et al., 2016).

Since HBcAg is the main target of the host immune response, therefore, mutations in the preC/C region could induce persistent HBV infection (Yim et al., 2015). M2RR HBcAg mutations leading to immune escape may lead to persistent HBV infection associated with disease progression (Wu et al., 2014). In addition, mutations such as I97F/L and P135Q, which inhibit the formation of the core nucleocapsid, may also play a role in disease progression by evading host innate immunity (Kim et al., 2012).

### 4.4. P mutations

Mutations in HBV polymerase regions are often associated with antiviral resistance. The emergence and selection of resistance mutations to nucleos(t)ide analogs in the P region, create a viral survival advantage and are the main barrier to successful anti-HBV NA therapy. In addition, mutations in HBV polymerase can also lead to amino acid changes in the HBsAg due to overlapping ORFs in the genome that allow the viral escape (Mokaya et al., 2018; Nguyen and Van Le, 2023). In a longitudinal study of CHB patients resistant to lamivudine, rtA181T mutation in the polymerase gene increased the risk of HCC occurrence. In addition, a large proportion of patients carrying the rtA181T mutation had the sW172* nonsense mutation, which result in truncated pre-S/S proteins (Yeh et al., 2011). The higher oncogenic potential of rtA181T/sW172stop mutant has also been reported in NIH3T3 cells (Lai and Yeh, 2008).

Considering that the incidence of HCC was higher in mice with a transgene encoding the RT region of HBV pol than in controls, there may be a relationship between the RT region and the HCC development by activating pro-apoptotic and pro-inflammatory responses. Also, the incidence of HCC in HBV patients with rtI224V or rtM309K mutation is 2.75 or 3.57-fold higher than in patients without each mutation, respectively (Chung et al., 2018). However, more studies are needed to determine the effects of HBV pol on HCC. In Figure 2A we summarized the main impacts of ORFs mutations on HBV which could lead to a higher risk of HCC.

## 5. HBV mutations as a HCC biomarker

Various studies have suggested that some HBV mutations could serve as potential prognostic biomarkers for HCC (Chaturvedi et al., 2019; Ge et al., 2019).

According to the results of the meta-analysis study, G1896A, A1762T, G1764A, and A1762T/G1764A mutations have been suggested as potential biomarkers to predict the occurrence of HCC (Wei et al., 2017). C1653T mutant is more prevalent with the progression of CHB from asymptomatic HBsAg carrier status to cirrhosis or HCC, indicating the accumulation of the mutation before the HCC diagnosis. Therefore, the C1653T mutation may be used as a useful prognostic biomarker of HCC (Liu et al., 2009). In addition, the increased effect of T1753V mutant and high serum level of AFB1-lysine adduct, which is the most reliable biomarker in monitoring long-term exposure to AFB1, on the HCC risk has been reported (Xu et al., 2010). HBx5 mutation is suggested as a risk factor for HCC progression, it could also serve as a potential biomarker to predict clinical outcomes of CHB individuals (Lee et al., 2011).

HBV mutations are not only associated with hepatocellular carcinoma but may also be associated with HCC survival including A1762T/G1764A mutations, which are predictive of postoperative survival in HBV-related HCC individuals (Xie et al., 2014). Also, TA combined with other core promoter mutations have been proposed as two promising diagnostic biomarkers for postoperative survival in HCC patients (Chen L. et al., 2016).

## 6. Conclusion and future perspectives

Epidemiological studies and some functional evaluations have shown the possible role of HBV mutations in increasing the risk of HCC. However, the causal relationship between HBV mutations and HCC has not yet been conclusively proven because there have been limited prospective longitudinal HBV cohort studies. Also, the molecular mechanism associated with HBV genome variations that modify carcinogenesis remains unclear. HBV variations may increase the risk of HCC indirectly by acquiring features that lead to increased immune evasion, viral replication or dysregulated host signaling pathways. Further prospective epidemiologic studies, in addition to molecular investigations of complete HBV genome sequences and evaluation of the host immune response to genotype-specific peptides, would be very helpful in defining this association.

A more complete understanding of the role of HBV genome variations in increasing the risk of HCC can lead to the appropriate management of patients. On the other hand, the discovery of new therapeutic targets and the development of more effective strategies for HCC treatment may be facilitated by investigating the commonly dysregulated pathways in HBV-related HCC. In addition, some HBV variations as prognostic biomarkers play an important role in the timely diagnosis and treatment of HBV-related HCC patients.

## Author contributions

SHSH and SRM decided and planned on the manuscript. SHSH, SRM, SMH, AH, and AG searched and reviewed the literature and drafted the manuscript. SRM and AG critically revised the manuscript for intellectual content. All authors read and approved the manuscript.

## Conflict of interest

The authors declare that the research was conducted in the absence of any commercial or financial relationships that could be construed as a potential conflict of interest.

## Publisher’s note

All claims expressed in this article are solely those of the authors and do not necessarily represent those of their affiliated organizations, or those of the publisher, the editors and the reviewers. Any product that may be evaluated in this article, or claim that may be made by its manufacturer, is not guaranteed or endorsed by the publisher.
